# Identifying, Engaging, and Supporting Care Partners in Clinical Settings: Protocol for a Patient Portal–Based Intervention

**DOI:** 10.2196/66708

**Published:** 2025-03-04

**Authors:** Catherine M DesRoches, Deborah Wachenheim, Jessica Ameling, Aysel Cibildak, Nancy Cibotti, Zhiyong Dong, Alexandra Drane, Isabel Hurwitz, Jennifer Meddings, Jody Naimark, Kimberly O'Donnell, Christine Winger, Sarah Stephens Winnay, Jordan Young, Jennifer L Wolff

**Affiliations:** 1 OpenNotes Department of Medicine Harvard Medical School Boston, MA United States; 2 OpenNotes Division of General Medicine Beth Israel Deaconess Medical Center Boston, MA United States; 3 Division of General Medicine Department of Internal Medicine University of Michigan Medical School Ann Arbor, MI United States; 4 ARCHANGELS Boston, MA United States; 5 BILH Primary Care Beth Israel Lahey Health Boston, MA United States; 6 Department of Medicine Veterans Affairs Ann Arbor Healthcare System Ann Arbor, MI United States; 7 Center for Clinical Management Research Veterans Affairs Ann Arbor Healthcare System Ann Arbor, MI United States; 8 Division of General Pediatrics Department of Pediatrics University of Michigan Medical School Ann Arbor, MI United States; 9 Department of Family Medicine Winchester Hospital Winchester, MA United States; 10 Beth Israel Lahey Health Primary Care Lexington, MA United States; 11 Division of Transplant Surgery Department of Surgery University of Michigan Medical School Ann Arbor, MI United States; 12 Department of Health Policy and Management Johns Hopkins Bloomberg School of Public Health Johns Hopkins University Baltimore, MD United States

**Keywords:** patient portal, previsit questionnaire, caregivers, care partners, questionnaires, support, engagement

## Abstract

**Background:**

In the United States, the landscape of unpaid care delivery is both challenging and complex, with millions of individuals undertaking the vital role of helping families (broadly defined) manage their health care and well-being. This includes 48 million caregivers of adults, 42 million of whom are caregivers of adults aged 50 years or older. These family care partners provide critical and often daily support for tasks such as dressing and bathing, as well as managing medications, medical equipment, appointments, and follow-up care plans.

**Objective:**

This study aimed to implement a novel patient portal–based intervention to identify, engage, and support care partners in clinical settings.

**Methods:**

The project team collaborated with 3 health care organizations (6 primary care practices in total) to design and implement a patient portal–based intervention. Three days in advance of a visit, patients were invited to log on to their patient portal account and answer a brief questionnaire as part of the routine electronic check-in process asking them to (1) identify themselves as the patient or someone answering for the patient, (2) report major life changes, (3) set the agenda for the upcoming visit, and (4) report on care partner responsibilities. Respondents’ answers to this brief questionnaire were available to providers ahead of the visit. Patients with care partner responsibilities, as well as care partners answering the questionnaire on behalf of patients, were provided a link to the ARCHANGELS Caregiver Intensity Index to measure the intensity of their caregiving role and motivate care partners to connect with suggested state and local resources.

**Results:**

The intervention was launched in September 2022 at Organization A. Organization B launched in May 2023 in one clinic and June 2023 in the other. In focus groups, staff and clinicians reported that the intervention was easy to implement and did not cause workflow disruption. At 6 months post implementation, across both organizations, a total of 22,152 patients had received questionnaires and 13,825 (62.4%) had submitted completed questionnaires. Full data will be reported at the completion of the intervention period.

**Conclusions:**

Early results suggest that the intervention could be an easily scalable and adaptable method of identifying and supporting care partners in clinical settings.

**International Registered Report Identifier (IRRID):**

DERR1-10.2196/66708

## Introduction

### Caregiving in the United States

In the United States, the landscape of unpaid care delivery is both challenging and complex. Millions of individuals undertake the vital role of helping someone else manage their health care and well-being. This includes 48 million caregivers of adults, 42 million of whom are caregivers of adults aged 50 years or older [1]. Family (broadly defined) care partners provide critical and often daily support for tasks such as dressing and bathing, as well as managing medications, medical equipment, appointments, and follow-up care plans [2-4]. The role of a care partner can be fulfilling as well as challenging. Many care partners report a sense of purpose and fulfillment related to the care they provide, and some research suggests that caregiving has a protective health effect [5-8]. Other studies suggest that having caregiving responsibilities can also be correlated with poorer mental and physical health, finding substantial rates of chronic illness and poorer mental health over time [9-13].

Surveys of care partners taken during the COVID-19 pandemic noted particularly high levels of stress and adverse mental health symptoms, including anxiety, depression, and suicidality [10-12], possibly due to the reduced availability of services related to social distancing and closures by home care services and caregiver respite services [13]. A survey of more than 10,000 US adults conducted between late 2020 and early 2021 revealed significant mental health issues. A total of 70% of all care partners reported experiencing at least one adverse mental health symptom such as anxiety, depression, or suicidal thoughts, in comparison to 32% of respondents with no care partner responsibilities [10]. While the pandemic represented an extraordinary challenge to care partners, surveys conducted before the pandemic also found significantly higher rates of adverse mental health conditions among this population [14,15].

Providing care partners with psychosocial support has been shown to help both care partners and their care recipients [16,17]. However, identifying care partners and linking them to services remains challenging [18]. While care partners frequently interact with the health care system on behalf of patients, they often lack a way to access support for themselves [19,20]. A visit to a care recipient’s health care provider rarely includes a discussion of care partner stress. As well, adding time to discuss caregiving responsibilities and available resources to an already busy clinical visit is often not feasible [21]. Identifying care partners (both patients who are care partners and care partners of patients) before or during clinical visits and connecting them to resources, without adding to clinical work, could be beneficial to patients, care partners, and clinicians.

### Use of Patient Portals

Electronic health records (EHRs) and secure patient portals offer a ready opportunity to identify care partners in need of services. Most health care providers use EHRs and have online patient portals through which their patients can access their medical information, message their clinicians, renew prescriptions, schedule appointments, and more [22]. Previsit questionnaires, administered through the patient portal in preparation for a visit, have become more commonplace [23]. These questionnaires can provide an opportunity for clinicians to learn more about patient needs and concerns before a visit [24]. In addition to previsit data collection, portals offer the opportunity for interventions and the provision of resources outside of the clinic visit [25]. Studies suggest that portal-based interventions can lead to improvements in psycho-behavioral outcomes, such as health knowledge, self-efficacy, and decision-making [25,26]. While these benefits are typically focused on patients’ reported health care needs, they can also be extended to other aspects of patients’ lives, including their caregiving responsibilities.

Here we report on a multisite intervention designed to implement a method of identifying, engaging, and supporting care partners in office-based clinical settings using the patient portal. Specifically, we sought to create a model of care that identified care partners through a previsit questionnaire ahead of their own health care appointments, engaged them through the incorporation of their self-reported visit concerns and priorities into the questionnaire data, and activated them to understand their caregiver intensity and connect with resources without requiring additional action from the care team. We report here on the intervention design and interim feasibility data. The intervention took place for 1 year and the interim data were gathered at 6 months.

## Methods

### Intervention Components

#### Caregiver Intensity Index

The ARCHANGELS Caregiver Intensity Index (CII) [27] is designed as a self-assessment instrument of caregiver intensity across multiple dimensions. Individuals who complete the CII are provided with a numeric score between 0 and 100, a color (clear: low intensity; yellow: moderate intensity; and red: high intensity), and the top two drivers and top two buffers of their caregiver intensity. Those completing the CII are then directed to a microsite with resources specific to their needs and the community in which the patient receives care. The project team collaborated with each implementing site to ensure that the resource page was comprehensive.

#### In-Clinic Materials

Each participating site was provided with in-clinic posters, wallet-sized handouts (referred to as “Care Cards”), and tip sheets for clinicians and staff. The posters and Care Cards included scannable QR codes that linked patients to the CII. These QR codes were specific to each site and modality, which allowed tracking of how resources were accessed (whether via poster, Care Card, after-visit summary, or previsit questionnaire). The tip sheets for clinicians and staff contained information on how to talk with patients about caregiving responsibilities and how to find patients’ completed questionnaires in the EHR.

#### Previsit Questionnaire

The team at Organization A convened regularly with the project team to draft and refine the previsit questionnaire, adapting a previously tested questionnaire [24] with input from clinic staff and clinicians as well as feedback from patient and family advisors in the organization’s Patient and Family Advisory Council. The final previsit questionnaire was also tested at Organizations B and C.

**Figure 1:**
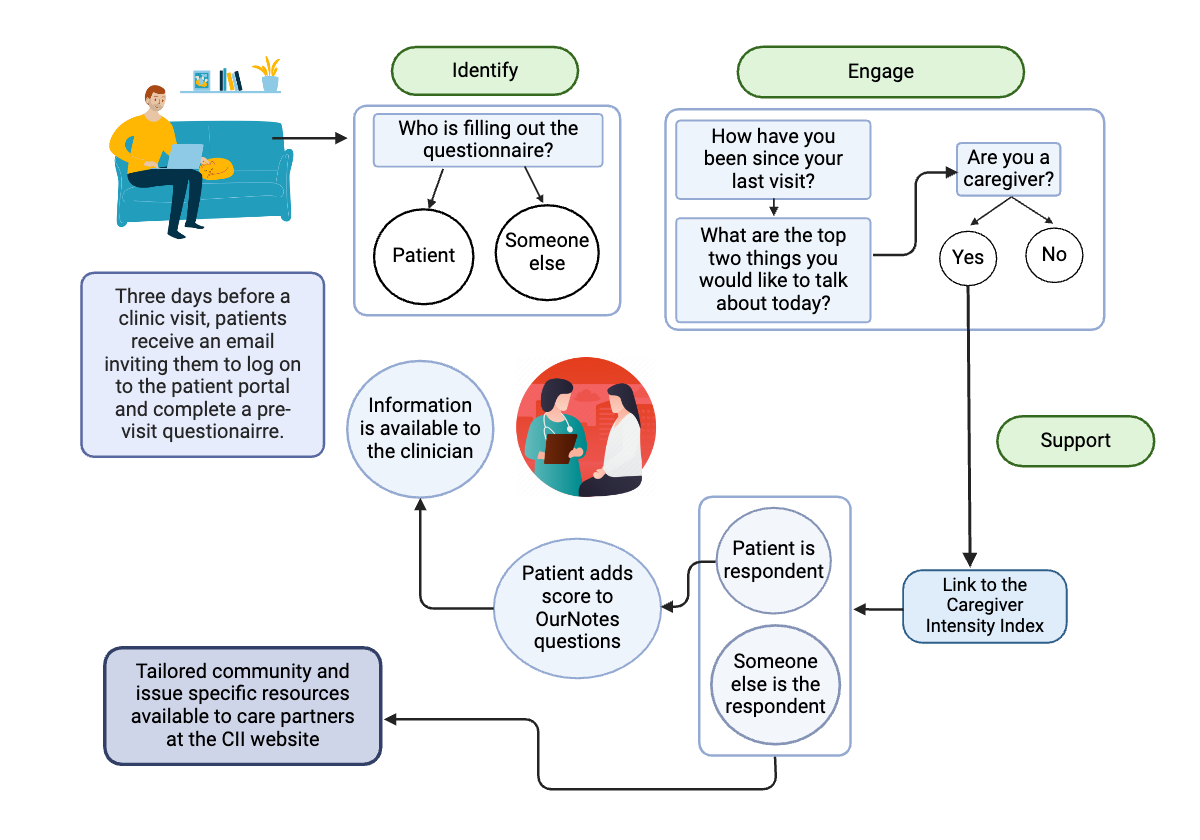
Previsit questionnaire flowchart.

The final previsit questionnaire, to be sent to all patients aged 18+ years with an upcoming visit (except those whose upcoming visits were Medicare annual wellness visits, annual physicals, or telehealth visits), comprised the following items (see Multimedia Appendix 1 for complete survey branching logic and the exact wording of the questions): (1) Identification: respondents were prompted to specify if they were the patient or were completing the previsit questionnaire on the patient's behalf (either as a designated portal proxy or by logging in as the patient; note: if someone other than the patient is filling out the questionnaire, they are answering the questions for the patient, not for themselves). (2) Life changes notification: respondents were asked to communicate significant life changes since their last clinic visit. (3) Visit agenda-setting: respondents were asked to identify the one or two most important things they wished to discuss at the upcoming visit. (4) Care partner responsibilities: respondents were asked if they had caregiving duties and to share for whom they provided care. (5) Receipt of care partner support: respondents were prompted to indicate if they received help managing their health and health care from another person. (6) Caregiver intensity index: respondents reporting caregiving responsibilities were given a link to the CII and provided with an opportunity to report their color and score in the previsit questionnaire. Those who answered the previsit questionnaire on behalf of the patient were given the CII link but were not given the option of sharing their score (Figure 1).

We integrated previsit questions into the EHR for testing, assessing both the patient-facing interface within the patient portal and the corresponding view for clinic staff and clinicians upon submission of responses.

#### EHR Macros

Two macros were developed: 1 for clinicians to incorporate information about the CII in the patient visit summary and another enabling clinicians to integrate questionnaire responses into their notes.

#### Education for Clinicians and Staff

Before implementation, the project team conducted sessions with staff and clinicians from participating practices to demonstrate how to access the previsit questionnaire within the EHR, identify workflow adjustments, and provide an overview of the in-clinic materials. The project was approved by the institutional review boards at all project sites.

#### Stakeholder Input

The project team met with staff and clinicians at each participating site at 3 and 6 months, both in person and virtually. These meetings were held to identify and address implementation issues, provide ongoing education and support to clinic staff and clinicians, and surface feedback and concerns about the initiative.

#### Plans for Statistical Analysis

Upon the intervention period’s completion at all sites, we will summarize the study participants’ demographics, their usage of the CII, and the Caregiver Intensity scores. To compare the characteristics between groups, we will use the chi-square test or Fisher exact test for categorical variables and the Student *t* test for continuous variables. Multivariable regression models will be used to explore the associations between the Caregiver Intensity score and patient demographic factors. We will also use qualitative analytic methods to identify key themes and subthemes in the free-text responses. All statistical analyses will be performed using SAS software (version 9.4; SAS Institute Inc).

### Ethical Considerations

The protocol was approved and deemed exempt from full review by the institutional review boards at Beth Israel Deaconess Medical Center (IRB protocol # 2021P000928) and the University of Michigan (IRB protocol # HUM00225451). Per the exempt status of the intervention, informed consent was not required. Patients were informed about their right to opt out of the intervention in the invitation email. All data will be deidentified in the analytic data file. No personally identifying information will be included in the analytic file. Participants were not compensated.

## Results

We engaged 3 health care organizations to collaborate on the design and implementation of the intervention (Figure 1). The first site (Organization A) is a prominent academic medical center network in the Northeast. Within this network, we partnered with three clinics situated in suburban areas outside a major metropolitan hub. Our second partner (Organization B) is a large academic medical center based in the Midwest. Within this center, we collaborated with two primary care clinics: a general internal medicine practice and a combined general internal medicine and pediatrics practice. Our third partner organization (Organization C) is a nonprofit health care entity serving primarily urban communities in the Northeast. The clinic associated with this organization predominantly serves low-income and racially diverse populations.

Two of these organizations are situated within regions commonly supported by the Ralph C Wilson Jr Foundation (the project funder), namely western New York and southeastern Michigan. The selection of the third site was based on existing strong professional relationships between project team members and the organization’s leadership, coupled with the organization’s keen interest in participating. Characteristics of participating health care organizations are shown in Table 1.

**Table 1:** Site characteristics.

Characteristics			Organization A		Organization B		Organization C
Total number of clinicians			Clinic 1: n=2 Clinic 2: n=6 Clinic 3: n=5		Clinic 1: n=22 Clinic 2: n=20		2
Total number of clinic staff			Clinic 1: n=17 Clinic 2: n=13 Clinic 3: n=15		—^a^		11
Clinic location			Clinic 1: suburban Clinic 2: suburban Clinic 3: suburban		Clinic 1: mixed Clinic 2: mixed		Urban
**Organizational payer mix, %**							
	Medicaid	14		28		57	
	Medicare	10		30		21	
	Private commercial insurance	72		40		20	
	Uninsured	2		2		1	
	Other unknown	3		—		—	

^a^Not applicable.

Each organization established a dedicated work group that convened regularly with the research team (Table 2). These groups consisted of clinical champions, clinic leadership, project managers, and information technology staff. All partner organizations use the same electronic health record system.

**Table 2:** Organizational workgroups.

Collaborators	Organization A	Organization B	Organization C
Clinical champion	✓	✓	
Project manager		✓	
EHR^a^ medical director	✓		
EHR team member	✓	✓	✓
Director of grants and program management			✓
Chief innovation officer	✓		
Social worker			✓
Practice administrator			✓

^a^EHR: electronic health record.

Organization A launched the intervention on September 30, 2022, and Organization B launched on May 17, 2023, in one clinic and June 1, 2023, in the other. Organization C was not successful in implementing the previsit questionnaire in their EHR and withdrew from the initiative. This was due to a mix of factors, including the lack of a clinical champion, the low usage of the patient portal among its patients combined with a higher reliance on paper documents by staff, and varied methods across clinics and staff as to if and how questionnaires submitted from the portal are identified and reviewed ahead of visits.

Adult patients at least 18 years of age were eligible to participate in the initiative. As shown in Figure 1, patients with a patient portal account and with an upcoming clinic visit were sent an invitation through the patient portal, 3 days ahead of a visit, to complete the previsit questionnaire. All visit types were included in the initiative with the exception of Medicare annual wellness visits, annual physicals, and telehealth visits. Annual wellness visits and physicals were excluded by request at participating sites because patients were already asked to complete a substantial number of questionnaires before those visits. Telehealth visits were excluded due to clinician concern that asking patients to fill out the previsit questionnaire would serve as a barrier to the telehealth login process and cause a delay in care. As shown in Figure 1, all the information provided by patients, or by others on patients’ behalf, through the previsit questionnaire was available to clinicians in the EHR. Patients and care partners who did not complete the previsit questionnaire could access the CII through QR codes on the in-clinic materials (eg, care cards, posters, or the after-visit summary).

At the 3- and 6-month check-ins, clinic staff and clinicians reported few challenges with implementing the intervention. The primary challenge was learning to access patients’ previsit questionnaire responses in the clinician and staff EHR view. Clinic staff and clinicians reported that some patients expressed appreciation for the opportunity to share and discuss their caregiving responsibilities. Further, they reported that few patients complained about being asked to fill out the previsit questionnaire. Clinic staff and providers said they felt better prepared to discuss care partner responsibilities with patients because they had resources to offer. Based on clinician and staff concerns with lengthy patient responses to the life changes and agenda-setting questions, the project team made minor wording changes to those questions and instituted character limits on the free text responses (see final questionnaire in Appendix). Finally, staff reported minimal disruption to workflows after adjustments to reduce the character limits of agenda-setting questions.

At 6 months post implementation, 25,611 surveys were assigned to 13,299 patients at Organization A and 16,265 questionnaires to 8853 patients at Organization B. At Organization A, 7076 questionnaires were received from 5350 patients. At Organization B, 6749 questionnaires were received from 4982 patients. Because the previsit questionnaire was tied to clinical visits, patients with multiple visits during the observation period received more than one invitation to complete a previsit questionnaire (Table 3).

Data for the full intervention period will be published in a future paper.

**Table 3:** Questionnaires assigned and submitted.

Disposition of questionnaires	Organization A	Organization B
Number of questionnaires assigned to patients, n	25,611	16,265
Number of questionnaires submitted by patients, n/n (%)	7076/25,611 (27.6)	6749/16,265 (41.5)
Number of patients assigned at least one questionnaire, n	13,299	8853
Number of patients submitted at least one questionnaire, n/n (%)	5350/13,299 (40.2)	4982/8853 (56.3)

## Discussion

### Principal Findings

We report on the rationale, development, and early uptake from the first 6 months of implementing a practice-based intervention aimed at identifying, engaging, and supporting care partners in office-based clinical settings using the patient portal. Early results from this work suggest that the intervention is acceptable to clinicians and staff and simple to implement. While we did not directly ask patients and care partners about their willingness to complete the previsit questionnaire, our robust response rate suggests that the intervention is feasible for patients and that it is possible to identify care partners before or during a clinical visit and connect them to resources.

To our knowledge, our intervention represents the first systematic attempt to identify, engage, and support care partners in clinical settings through a previsit questionnaire. Previous work focused on identifying patients’ visit priorities using a similar methodology and found much lower rates of patient uptake than our preliminary findings suggest [24]. Understanding the extent to which the local clinical environments and patient populations affected implementation will be a critical area of focus for the final evaluation [28]. Our preliminary findings suggest that patients were generally receptive to completing the previsit questionnaire through the patient portal. The usage of previsit questionnaires is becoming more prevalent for assessing Social Determinants of Health and identifying resources or referrals that may benefit patients [29]. Incorporating caregiver-related responsibilities within Social Determinants of Health assessment efforts could also merit attention as a strategy for raising awareness of caregiver needs and integrating care partner identification and support within care delivery if our findings hold in the final evaluation.

While the role of a care partner can be fulfilling, it can also be fraught with numerous challenges, leading to elevated rates of mental and physical health issues among care partners [28]. Identifying care partners is a first step in understanding their capacity and needs, and in facilitating access to appropriate resources. Doing so within care delivery enables clinicians to become aware of caregiving responsibilities; however, busy clinical practices may struggle to address these needs. By providing resources outside of the clinical visit, our intervention could help to support caregivers, clinicians, and staff. Staff reported minimal burdens and disruptions to their workflow and the intervention was relatively simple to implement.

Our protocol has several limitations that should be noted. First, while the protocol includes in-office materials for patients and clinicians, patients must have access to the internet in order to take the CII and explore the resources offered. This requirement may exclude a subset of patients with lower digital literacy. Future efforts could explore the possibility of, for example, text-based options using mobile devices. Second, our protocol is implemented in 5 primary care practices associated with academic medical centers. These centers may have resources for implementation that are not available in community-based practices. Finally, our protocol was designed for and implemented in health care organizations with the same commercially available EHR system. Implementing in other EHR systems may require adjustments to the protocol.

In the final 6 months of the project, we will monitor whether staff continue to report minimal workflow disruptions, whether patient feedback remains predominantly positive or neutral, or if there is an increase in complaints. If current results hold, and we find that patients with caregiving responsibilities are using the CII, this portal-based intervention could be a scalable and adaptable method of identifying, engaging, and supporting patients and their care partners in clinical settings. Should that be the case, we will widely disseminate our findings through the peer-reviewed and gray literature, as well as develop and disseminate “how-to” toolkits for primary care practices. Further studies focused on implementation in a larger number of diverse settings, by patient population, electronic medical record vendor, types of care delivery organizations, and geographic regions, will also be important as we seek to engage and support care partners through the health care system.

### Conclusion

Millions of adults help another person with their health and care, and their needs are often unidentified and unmet. Despite the impact of caregiving responsibilities on physical and mental health, often their own health care providers do not know about their responsibilities. The early assessment of this unique pilot seeking to identify care partners through previsit questionnaires and in-office materials indicates that this low-burden effort may be an effective tool for identifying and supporting care partners. Future analyses of data from the full one-year pilot will give a more complete picture of the results and the possibility of adapting and scaling this intervention beyond the individual clinics involved in the study.

## Appendix

Multimedia Appendix 1 Previsit questionnaire.

## Abbreviations

CII: Caregiver Intensity Index
EHR: electronic health record

## Data Availability

The datasets generated during and analyzed during this study are not publicly available per the requirements of the participating organizations to maintain strict confidentiality and disclosure standards for patent data obtained during the course of routine care. For this reason, the data are also not available from the corresponding author.
