# Supplementary material for: Identifying, Engaging, and Supporting Care Partners in Clinical Settings: Protocol for a Patient Portal–Based Intervention
Source: JMIR Res Protoc. 2025 Mar 4;14:e66708. doi: 10.2196/66708 (PMC11920659; doi:10.2196/66708)
Supplement: Multimedia Appendix 1 [file resprot_v14i1e66708_app1.docx]

Text for patients and care partners

Questions for patients

Questions for care partners

**OurNotes for Care Partners**

**Epic Questionnaire**

To get ready for your visit, please tell us how you’ve been doing since we last saw you. We would also like you to tell us if you are helping someone else with their health and well-being. Helping another person in that way can be rewarding but also stressful, and we have resources that can help.

This questionnaire is not for any urgent issues; if something is urgent, you need to call the office or 911 as appropriate.

Q1. Are you the patient or someone responding for the patient?

I am the patient (go to Q2)

I am filling out the questionnaire for the patient. [Go to Q7]

Q2. Since the last time we saw you, have you had any major change in your life or anything else that you would like us to know (limit to 200 characters)?

Q3. For your upcoming visit, what are the one or two most important things you would like to discuss with us? (80 characters)

Q4. Caring for the health and wellness of someone else can be rewarding AND really hard. This can include helping with personal needs, chores, healthcare, or managing money. You might drive someone to doctor’s appointments or check in regularly to see how they are doing or provide other kinds of help. Is this something you do?

Yes

No

Q5. Do you have someone in your life that is helping with your health and well-being other than medical professionals?

Yes

No

Q6. [IF q4 = Yes] Please tell us, who are you helping? Select all that apply.

A parent

An adult child

A friend

A neighbor

Someone else [please specify]

Q7. [If Q1 = filling out the questionnaire for the patient] What is your relationship to the patient?

Spouse

Child

Sibling

Friend

Paid caregiver

Someone else

Q8. Since the last time we saw the patient, have they had any major change in their life or anything else that they would like us to know (limit to 200 characters)?

Q9. For the upcoming visit, what are the one or two most important things the patient would like to discuss with us? (80 characters)

Q10. Caring for the health and wellness of someone else can be rewarding AND really hard. This can include helping with personal needs, chores, healthcare, or managing money. You might drive someone to doctor’s appointments or check in regularly to see how they are doing or provide other kinds of help. If you are helping the patient in this way, we’d like to know and to connect you to resources that may help. Is this something you do?

Yes

No

[if Q4 OR Q10= yes] You answered that you are caring for someone else. To understand how this is affecting you, you can take a 2 minute Caregiver Intensity Index quiz (CII). The CII is a quick, completely confidential quiz that takes less than 2 minutes and gives you a score with a corresponding color – red, yellow, or green. Your score will tell you where things seem to be going well and where you could probably use more support. The CII then directs you to resources that may help.

Click this link to take the quiz – you can write down your score and color, print them to bring to your next appointment, or share them with your provider below.

Q11. Would you like to share your score and color with the provider?

Yes

No, I do not want to share it

I did not take the index quiz.

Q12. Caregiver Intensity Index Score and Color

Q13. [If Q10 = 1] Did you take the quiz?

Yes

No
